# Acid Rain and Flue Gas: Quantum Chemical Hydrolysis of NO_2_

**DOI:** 10.1002/cphc.202200395

**Published:** 2022-08-16

**Authors:** Filipe Menezes, Grzegorz Maria Popowicz

**Affiliations:** ^1^ Institute of Structural Biology Helmholtz Zentrum München Ingolstädter Landstr. 1 85764 Neuherberg Germany

**Keywords:** ab initio calculations, atmospheric chemistry, thermochemistry, thermodynamics, COSMO-RS

## Abstract

Despite decades of efforts, much is still unknown about the hydrolysis of nitrogen dioxide (NO_2_), a reaction associated with the formation of acid rain. From the experimental point of view, quantitative analyses are hard, and without pH control the products decompose to some reagents. We resort to high‐level quantum chemistry to compute Gibbs energies for a network of reactions relevant to the hydrolysis of NO_2_. With COSMO‐RS solvation corrections we calculate temperature dependent thermodynamic data in liquid water. Using the computed reaction energies, we determine equilibrium concentrations for a gas‐liquid system at controlled pH. For different temperatures and initial concentrations of the different species, we observe that nitrogen dioxide should be fully converted to nitric and nitrous acid. The thermodynamic data in this work can have a potential major impact for several industries with regards to the understanding of atmospheric chemistry and in the reduction of anthropomorphic pollution.

## Graphical abstract

**High‐level quantum chemistry** and state‐of‐the‐art solvation models are used to determine the temperature dependent Gibbs free energies for a network of reactions involving the hydrolysis of nitrogen dioxide. The equilibrium for two‐phase systems is investigated, and implications for atmospheric chemistry are assessed.

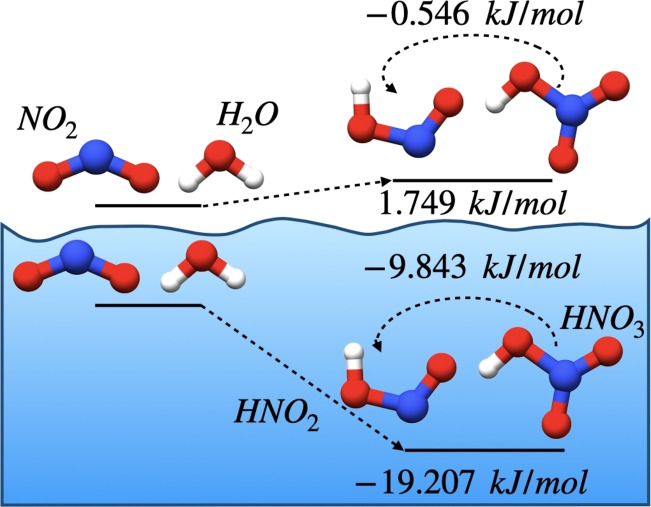

## Introduction

It has been acknowledged for several decades that acid rain and other environmental issues have an anthropomorphic origin.[^1^, ^2^, ^3^] It is estimated that two thirds of sulfur oxides (SO_x_) and one fourth of nitrogen oxides (N_x_O_y_) are produced in the generation of electricity from of fossil fuels, and several other industries have been pointed out as further contributors to the production of those pollutants.

**Figure 1:**
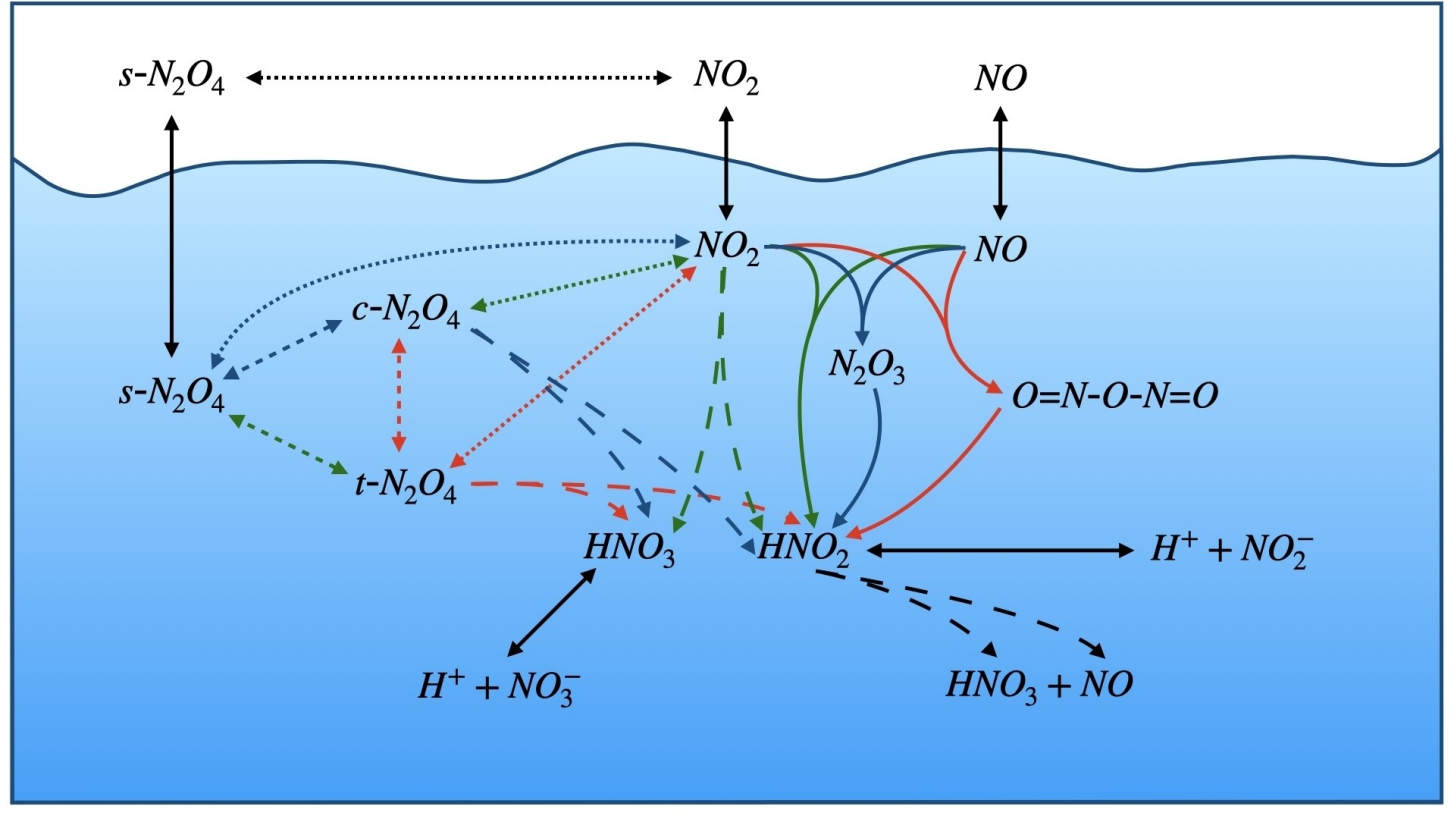
Schematic for the complete system of reactions studied.

When released to the atmosphere, these gases react with water resulting in several acids in gaseous and particulate forms. Upon accumulation in cloud water, the latter precipitate in the form of acid rain. The effects of acid rain are manifold,[^4^, ^5^] for instance degradation of human patrimony and the deterioration of soil and freshwater ecosystems by modification of their chemical composition. The latter may take such proportions that aqueous ecosystems may become unsuitable for sustaining varied lifeforms.

The Clean Air Act^[6]^ was a major contribution for controlling static and ambulant sources of air emissions, particularly the ones related to SO_x_. The regulation of N_x_O_y_ began later,^[7]^ so that these remain a severe problem that must be tackled.

Nitrogen dioxide, NO_2_, is one of the most problematic members of the family of N_x_O_y_. By means of hydrolysis, it is accepted to be the major contributor for the formation of nitrous and nitric acids in the atmosphere.[^3^, ^8^] The latter, nitric acid, was furthermore associated with the springtime ozone hole.^[9]^ When in the presence of amines, this reddish‐brown gas yields highly carcinogenic nitrosamines,[^10^, ^11^] which are also tightly regulated. However, NO_2_ also plays an important role for human society, since it is an important intermediary in the industrial synthesis of nitric acid,^[12]^ of paramount relevance to produce fertilizers.

The reaction of NO_2_ with water has been studied for over a century, yet, despite decades of effort, the reaction's mechanism is not entirely clear.^[13]^ From the experimental point of view this is hindered by 1) the formation of stable fogs and condensates; 2) the reactions’ rate, which strongly depends on conditions; 3) the existence of alternative pathways that regenerate NO_2_.[^8^, ^14^, ^15^, ^16^, ^17^] The violence of this reaction makes it furthermore extremely hard to measure virtually any physical or thermodynamical property for the system, so that, *e. g*., a search for the Henry constant of NO_2_ in water over different databases barely leads to any result.[^18^, ^19^] Perhaps the most accurate measurement to date of Henry constants comes from the work of Lee and Schwartz.^[20]^ These authors tabulate however a single value at 22 °C.

Irrespective of the application, complex networks of reactions have been proposed, which are often also based in broadly estimated and inaccurate data (*c.f*. Figure 1 for the network herein considered). In this work, the equilibrium thermodynamics of the hydrolysis of nitrogen dioxide is studied using Coupled Cluster theory with a full treatment of Singly and Doubly excited configurations, as well as with a perturbative treatment of Triple excitations (CCSD(T)). In order to avoid the limitations of finite size atomic basis sets, we extrapolate our results to Complete Basis Set (CBS). Coupled Cluster has been shown to be one of the few single‐reference *ab initio* methods with the ability to accurately describe the complex electronic structure of the species involved in this system.^[21]^ Other wavefunction and DFT methods have been employed by others, though these are not reliable enough for general application on NO_2_ and related molecules. These methods also lack the required accuracy for generating high‐level thermodynamic data.^[21]^

Solvation corrections are obtained by means of the COnductor like Screening MOdel for Real Solvents (COSMO‐RS). COSMO‐RS uses quantum chemical molecular charge densities to calculate chemical potentials and other properties of molecules in solution. This is a very high‐level method, which proved several times to deliver extremely accurate chemical potentials in solution.[^22^, ^23^] The CCSD(T)/COSMO‐RS results here presented yield state of the art thermodynamics in gas phase and in liquid water for the NO_2_ system. For the sake of consistency, the data we calculated is compared against the best experimental observations we collected. With this information, a two‐phase reactive equilibrium is solved to determine the concentrations of the most relevant species according to several conditions.

Our work will help to better understand the behavior of this important pollutant in gas phases and in liquid water. This, in turn, will lead to better design of industrial gas treatment facilities, further reducing NO_2_ pollution.

## Results and Discussion

**Figure 2:**
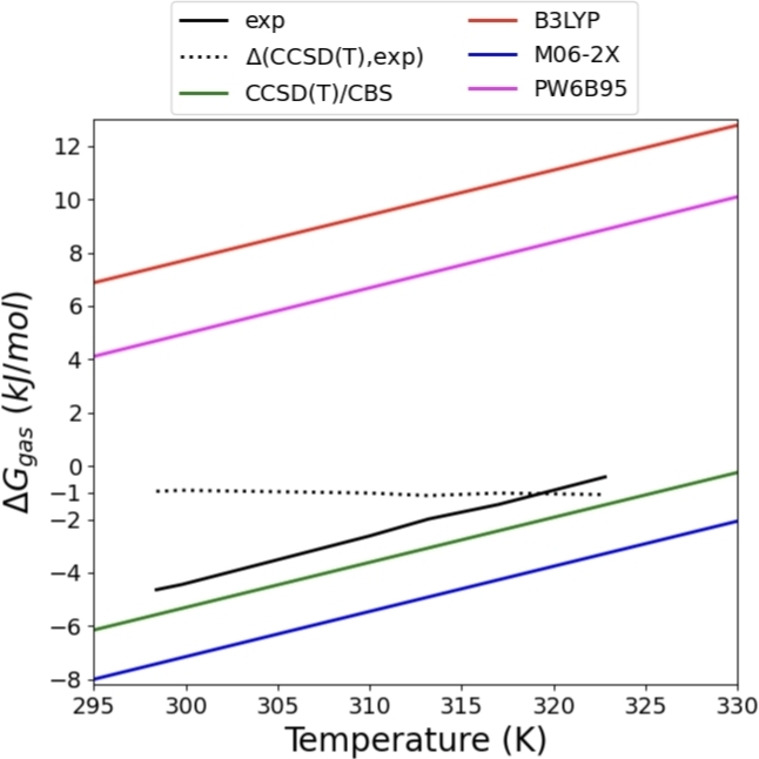
Example benchmark showing the superiority of CCSD(T) against several DFT methods for the dimerization of NO_2_ in the gas phase. Comparing CCSD(T)/CBS data against several density functionals (using the basis set def2‐QZVPP) and experimental data taken from [24].

Gibbs free energies in gas phase and in an aqueous solution for the two most relevant processes in the hydrolysis of NO_2_ are given in Table 1 and Figure 2. The complete list of temperature dependent thermodynamic data in both phases is provided in the supplementary material. Data is always provided in the form of fits, which are valid for the temperature range of 273.15–373.15 K. This means that the quantities in those equations should not be directly interpreted as enthalpies nor entropies. Gas phase enthalpies and entropies are given separately in another section of the supplementary material. Again, we provide linear fits based on the calculated data. Non‐linear terms have a minimal impact for the temperature range we studied, particularly for enthalpies. Unless otherwise stated, we discuss the data considering atmospheric pressure.

**Table 1:** Change in Gibbs free energies for the two most important reactions in defining the equilibrium state of NO_2_ hydrolysis. Equations fitted for the temperature range 274.15‐373.15 K.

	Reaction	ΔG (T) [kJ/mol]
1G	$2NO_{2}\left(g\right)+H_{2}O\left(g\right)\;\vec{\leftarrow }\;HNO_{3}\left(g\right)+HNO_{2}\left(g\right)$	0.145T-41.513
1 A	$2NO_{2}\left(aq\right)+H_{2}O\left(l\right)\;\vec{\leftarrow }\;HNO_{3}\left(aq\right)+HNO_{2}\left(aq\right)$	0.110T-52.093
2G	$3HNO_{2}\left(g\right)\;\vec{\leftarrow }\;HNO_{3}\left(g\right)+2NO\left(g\right)+H_{2}O\left(g\right)$	37.815-0.125T
2 A	$3HNO_{2}\left(aq\right)\;\vec{\leftarrow }\;HNO_{3}\left(aq\right)+2NO\left(aq\right)+H_{2}O\left(l\right)$	37.809-0.094T

Figure 2 shows a benchmark for the change in Gibbs free energy for the dimerization of nitrogen dioxide in the gas phase. All the DFT data is consistently calculated using quadruple zeta basis sets (def2‐QZVPP). This data is compared against CCSD(T)/CBS and experimental results.^[24]^ Of all DFT methods, only the Minnesota functional M06‐2X gets reasonably close to the *ab initio* and experimental data. Nevertheless, the deviations with respect to experimental data are still about 3 kJ/mol, which renders the M06‐2X data too inaccurate for studying equilibrium thermodynamics. Interestingly, the widely used density functional B3LYP even fails to predict the spontaneity of the reaction. The dotted line shows the deviation between CCSD(T)/CBS and the experimental data. This is an approximately constant line with value of 1 kJ/mol.

Durham *et al*.^[25]^ used literature values of kinetic constants to estimate the Gibbs free energies for the dimerization of NO_2_ in water. Their calculation results in the value of −27.5 kJ/mol at 25 °C, which deviates by 11.4 kJ/mol from our calculations. Using the values reported by Huie^[10]^ for the forward reaction, the Gibbs free energy for the same reaction lowers to −22.1 kJ/mol, which is closer to our results. Similarly, England and Corcoran^[14]^ report kinetic data that yields a Gibbs free energy for reaction 1A of −5.6 kJ/mol at 25 °C. This is 13.6 kJ/mol higher than ours. Saramaki and coworkers^[26]^ report however data in contradiction to England and Corcoran's. Consequently, we cautiously consider the accuracy and meaningfulness of this comparison.

**Figure 3:**
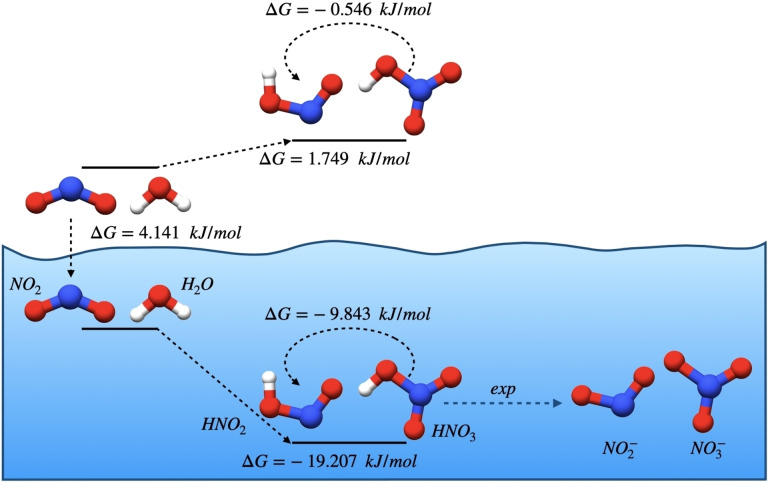
The main reactions considered in the equilibrium study and their respective (calculated) Gibbs free Energies at 298.15 K.

Further, we detail the thermodynamics of the reactions in Figure 1. To aid the reader, reference is made to the specific reactions and the tables in the supplementary material. The thermodynamics for the main reactions studied are summarized in Figure 3.

The dimerization of NO_2_ can form three main species,^[21]^ symmetric N_2_O_4_ (s‐N_2_O_4_), and two asymmetric conformers named trans (t‐N_2_O_4_) and cis (c‐N_2_O_4_). In the gas phase, given sufficiently large temperatures, NO_2_ is not expected to dimerize. The formation of c‐N_2_O_4_ stops being spontaneous at 140 K, t‐N_2_O_4_ at 225 K, and the symmetric isomer at temperatures above 332 K. Our values for the equilibria are in good agreement with the literature,[^21^, ^27^] and they differ by approximately 1 kJ/mol with respect to experimental values.^[24]^

When in water, all conformers of N_2_O_4_ are stabilized. Enthalpies for the formation of these species are lowered and in the case of asymmetric dimers there is furthermore a less penalizing entropic term. s‐N_2_O_4_ is still expected to be the dominant species for the whole temperature range of liquid water (*cf*. supplementary material). The relative stability of s‐N_2_O_4_ towards t‐N_2_O_4_ decreases however with temperature, such that we expect a difference of about 1.5 kJ/mol between t‐N_2_O_4_ and s‐N_2_O_4_ at water‘s boiling point. The curves for the formation and hydrolysis of c‐N_2_O_4_ are parallel to the respective data for t‐N_2_O_4_, with a shift to larger values. Based on the calculated data we may build “thought experiment” solutions of NO_2_ dimers in water. These are expected to be mainly composed by s‐N_2_O_4_ and to a lesser extent by the respective asymmetric forms. Expectedly, the weights of the latter species increase with temperature, though the relative stability of the trans and cis forms should remain approximately constant. At water‘s boiling point, the expected composition is approximately 5 % c‐N_2_O_4_, 35 % t‐N_2_O_4_ and 60 % s‐N_2_O_4_.

The gas phase hydrolysis of NO_2_ (the equivalent to green large, dashed arrows in the gas phase; 1G in Table 1) is non‐spontaneous for temperatures above 286 K. If we consider dimers of NO_2_, one observes that the gas phase hydrolysis of asymmetric conformers is favorable for the whole temperature range studied. For s‐N_2_O_4_ there is however a thermodynamic impediment, since the hydrolysis reaction is non‐spontaneous in gas.

Different authors proposed reaction pathways that connect t‐N_2_O_4_ and c‐N_2_O_4_ to the hydrolysis products in the gas phase.[^26^, ^27^] The main finding was that reaction barriers were lower for the former than for the latter. Furthermore, transition states involving s‐N_2_O_4_ were all energetically inaccessible. Taking these observations in consideration, then the gas phase hydrolysis of NO_2_ is hindered by 1) the kinetics of hydrolysis of t‐N_2_O_4_, 2) the unfavorable thermodynamics for forming the dimeric species and 3) high activation barriers for the formation of t‐N_2_O_4_.^[21]^

In the presence of water there is a shift of the calculated Gibbs free energies for lower values. The latter are negative for the whole temperature range studied, irrespective of the starting species (NO_2_ or any of its dimers). Though spontaneous, the hydrolysis of s‐N_2_O_4_ shows the incorrect temperature dependence,^[14]^ for which we conclude that the reaction must be kinetically unfavorable. The hydrolysis process is however spontaneous for any of the asymmetric conformers of N_2_O_4_ and these show the correct slope. Even though we did not optimize any transition state for this work, we may argue that from the statistical mechanical point of view, the solvation correction to the chemical potentials should be identical for t‐N_2_O_4_, c‐N_2_O_4_ or any of the respective transition states. The lowering of the activation barriers in solution for the formation of t‐N_2_O_4_ and c‐N_2_O_4_ should therefore be similar to the respective lowering of the reaction's Gibbs free energy. With our data it seems then plausible that conclusions for the gas phase reaction transpose also to aqueous solutions. The difference in behavior between the different media should be an effect of how the solvent stabilizes the different species.

The decomposition of nitrous acid (long dashed‐black arrows; 2G and 2A in Table 1) in water is favorable for temperatures higher than 403 K. HNO_2_ is therefore not expected to decompose in liquid water. The situation differs however in the gas phase, since the reaction is spontaneous for temperatures above 303 K. Irrespective of the phase, the main driving force for the reaction is the change in entropy.

Whenever HNO_2_ and nitrite are present in water (Gibbs free energies of solvation for nitrous acid and its pK_a_ are given in the supplementary material), then the gas phase decomposition of nitrous acid may be neglected altogether. This is particularly true for less acidic conditions, where nitrous acid is undissociated. In strongly acidic media the situation changes as there are large amounts of undissociated HNO_2_ in water and an equilibrium between two phases should be established. Though the decomposition of HNO_2_ is non‐spontaneous in water, the reverse reaction is feasible, which allows the conversion of nitric acid into nitrous acid. This reaction is therefore important to consider from the equilibrium point of view. Based on the description above, one should not expect that equilibrium concentrations of nitrate and nitrite (or the respective acids) match. In less acidic media, it is to anticipate that some nitrate is converted to nitrite, and in strongly acidic media the opposite should take place.

Association reactions between NO_2_ and NO to form N_2_O_3_ or ONONO (red and blue full arrows; 6G, 6A, 6G′ and 6A′) are not entropically favorable in any phase considered. The driving force is the change in enthalpy. These reactions are spontaneous in gas for temperatures below 337 K and 314 K, respectively. In water, the reactions remain spontaneous beyond water‘s boiling point (568 K) and 366 K (respectively). The reaction of any of N_2_O_3_ conformers with water to yield nitrous acid (8G, 8A, 8G′ and 8A′) is only possible in the gas phase at extremely low temperatures. For N_2_O_3_ itself, the threshold temperature is so low (5 K) that the reaction is for practical purposes never favorable. For ONONO, the reaction is feasible for temperatures up to 165 K. These reactions are thus not relevant for atmospheric processes. In the presence of liquid water, the conversion of N_2_O_3_ into nitrous acid is hindered by both enthalpy and entropy considerations. The decomposition of ONONO is however always spontaneous due to a strong enthalpic gain and an almost zero entropic penalty.

Finally, we observe the reactions of nitric oxide with nitric acid to form nitrogen dioxide and water (reactions 9G and 9A in table 1 of the supporting material). These reactions show in both phases an entropic driving force. For temperatures above 279 K, the gas phase reaction becomes spontaneous. In water, the reaction is never spontaneous. Therefore, except at sufficiently low pH values, in which undissociated nitric acid may exist, this reaction may be neglected. This is particularly true in pH‐controlled solutions.

Although most applications involve large cocktails of chemicals, we used the calculated thermodynamic data to understand the hydrolysis of nitrogen dioxide from the equilibrium point of view. A detailed description of the set of reactions selected for determining the equilibrium is described in the supplementary material, along with the respective reasoning for our choices. Other details relevant to the thermodynamic model are also provided.

**Figure 4:**
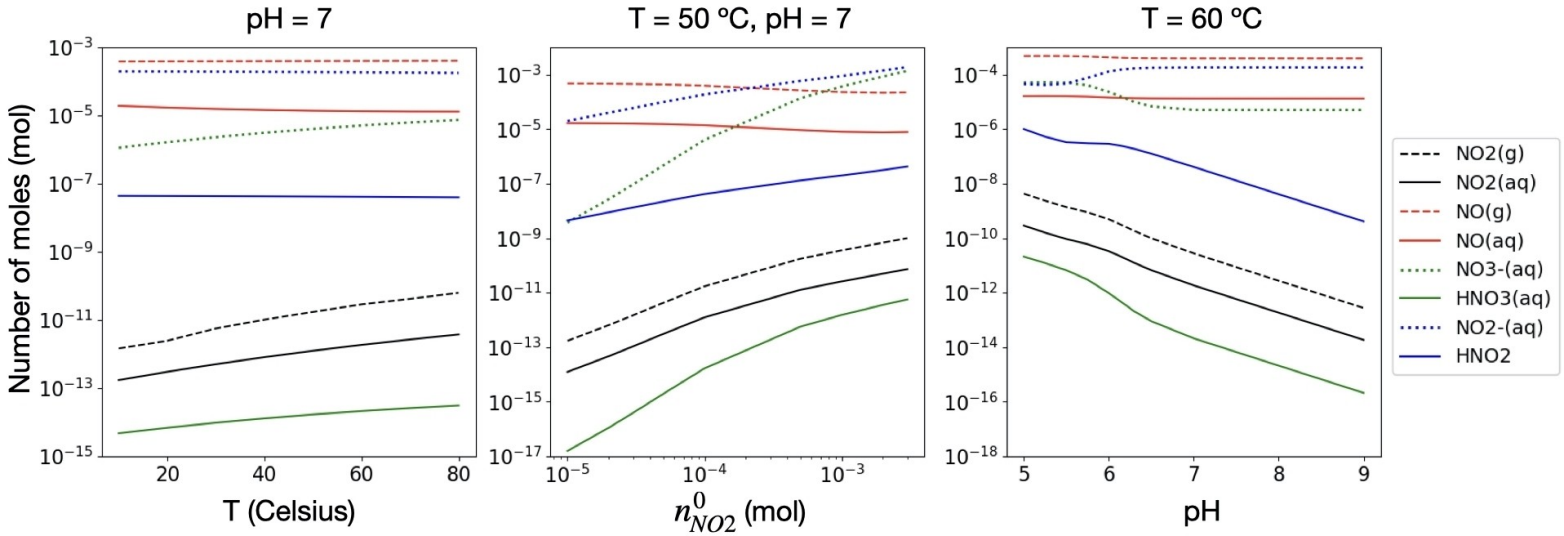
(Left) equilibrium number of moles of several species for systems at pH=7 with initially $10^{-4}$ mol of NO_2_, $5\times 10^{-4}$ mol of NO and 0.5 mol of water. (Middle) the temperature is fixed at 50 Celsius, pH=7 and the initial concentration of NO_2_ ($n_{NO2}^{0}$ ) is varied. The initial amount of NO and water is not modified. (Right) the equilibrium number of moles for several species at different pH values in systems at 60 Celsius and with initially $10^{-4}$ mol of NO_2_, $5\times 10^{-4}$ mol of NO and 0.5 mol of water.

Figure 4 shows the evolution of equilibrium concentrations for the most relevant species according to several conditions. We study 1) temperature effects at pH 7 and fixed initial concentration of nitrogen dioxide ($n_{NO2}^{0}$ ); 2) the effect of different $n_{NO2}^{0}$ at 50 Celsius and pH 7; 3) pH effects at 60 Celsius and fixed initial concentration of NO_2_.

A common feature of all the studies is the complete conversion of NO_2_ into nitrite and nitrate. Though there is a residual amount of nitrogen dioxide in the gas phase, the remaining concentration in water is even lower. Temperature effects are as expected from the thermodynamics of the main reaction: the consumption of NO_2_ at 10 Celsius is about one order of magnitude larger than at 80 Celsius. In practice, this is however irrelevant because the reaction is complete. The calculated values can be well understood using the definition of the respective equilibrium constants and a direct application of Le Chatelier's principle. HNO_3_ is a very strong acid. Consequently, this species is expected to shift the chemical equilibrium towards the products. The quadratic dependence of the equilibrium constant on the activity of NO_2_, guarantees the almost complete consumption of this species.

The pH effect on the equilibrium concentrations of NO_2_ is also the expected: since the reaction generates two acids, the yield of removal of the gas increases with the total pH of the system. This means that the higher the pH, the less NO_2_ will be available in any of the phases. Roughly estimated, one may say that one unit in pH corresponds approximately to a change of one order of magnitude in the equilibrium concentration of gaseous and aqueous NO_2_.

In agreement with our previous observations, the equilibrium concentrations of nitrite and nitrate differ for several of the conditions studied. This is due to the association reaction between NO and HNO_3_ to form nitrous acid (the reverse of the reaction with long dashed black arrows, or 2A in Table 1). Increasing the temperature brings the concentrations of nitrite and nitrate closer together, though the amount of nitrite is always one to two orders of magnitude larger than that of nitrate. Though temperature effects are rather weak, the pH may significantly affect the respective equilibria. At about pH=6.5 the concentrations of anions begin to converge and at pH=5.75 these are already matching. During the transition period, the concentration of HNO_2_ shows a plateau, whereas that of HNO_3_ changes slope. Furthermore, the equilibrium concentration of gaseous nitric oxide is also affected. This is not immediately visible due to its already high initial concentration and the logarithmic nature of the representation. The aqueous concentration of this gas is however unaffected. Other than this, NO is a rather inert species in the system and its solubility in water is well represented by Henry's law.

Increasing the initial concentration of NO_2_ decreases the gap in the equilibrium concentrations of anions. This is because the association of NO and HNO_3_ to form HNO_2_ has a cubic dependence on the latter, and it is quadratic on nitric acid. It thus leads to a less favorable ratio between the concentrations of products and reagents.

Atmospheric processes or even simulation of processes involving flue gas involve the presence of other species in the system, for instance SO_x_ or carbon dioxide. Though explicitly accounting for the effects of any of these components in our model system is beyond the scope of this work, we can infer indirectly on the influence that carbonic acid and its conjugate bases should have on the hydrolysis of NO_2_.

The increased presence of carbon dioxide typically acidifies water. Rainwater with carbonic acid might have pH values around 5.5,^[1]^ which is close to the lower limit of our study. Though the pH effect on the equilibrium concentration of NO_2_ is quite pronounced (based on the slope), the practical effect is rather weak. The yield of the hydrolysis reaction at pH=5.5 is approximately 30 times smaller than at neutral pH. The net effect of carbon dioxide is however to change the yield from 99.9999 % to 99.997 %. Even in the limit of rainwater at pH=5, the effect would be of no practical relevance (yield of 99.99 %).

On the other hand, in highly acidic media, nitrous acid decomposes to form nitric acid, which leads to the previously discussed overaccumulation of nitrate (or nitric acid). Because carbonic acid and its conjugate bases are efficient buffers, the presence of dissolved CO_2_ may keep the system from extreme acidification, thus hindering the over acidification of rainwater.

## Conclusions

In the present contribution, we studied the hydrolysis of nitrogen dioxide from the perspective of equilibrium thermodynamics, using high‐level quantum chemical methods and accurate solvation models. With complete basis set CCSD(T) data we calculated gas phase Gibbs free energies for a vast set of reactions of relevance for the formation of acid rain. With COSMO‐RS we determined corrections that allowed us to estimate temperature dependent thermodynamic data in aqueous phases. We verified that most processes are viable only in aqueous phase and except for a few decomposition reactions, the gas phase is reasonably inert. This is in excellent agreement with experimental observations. With this thermodynamic information, we calculated equilibrium concentrations for the main species at several conditions. These model scenarios are consistent with the experimental data available in the literature. The effects of dissolved carbon dioxide are analyzed based on pH effects on the hydrolysis reaction. The latter may be potentially beneficial by acting as a buffer, thus keeping the system's pH fixed.

This case‐study shows furthermore how high‐level quantum chemistry, in conjunction with accurate solvation models, may help complementing gaps in experimental data. CCSD(T) extrapolated to complete basis set can consistently capture in a qualitative and quantitative manner the intricate electronic structure of nitrogen dioxide and related species. With detailed understanding and high‐quality thermodynamics for one of the processes with major environmental impact, the data herein supplied may be useful for reducing pollution associated to, *e. g*., the automotive and chemical industries.

## Computational Details

Geometry optimizations were performed using the B3LYP^[29]^ functional with the def‐TZVP basis set as available from TmoleX 4.4.0 and TURBOMOLE 7.3.[^30^, ^31^, ^32^, ^33^] We found this the most suitable basis set in terms of the quality of the resulting equilibrium geometries and calculated vibrational frequencies.^[34]^ Energetics were improved using extrapolated complete basis set energies at the CCSD(T) level. We used the method of Halkier *et al*.^[35]^ for Hartree‐Fock energy extrapolation and the method of Helgaker *et al*.^[36]^ for correlation energies. CCSD(T) calculations using the augmented variants of Dunning basis sets[^37^, ^38^] were performed with ORCA 5.0.2.[^39^, ^40^, ^41^, ^42^] For the purpose of basis set extrapolation, we used the basis sets aug‐ccpVDZ, aug‐ccpVTZ and aug‐ccpVQZ. All CCSD(T) calculations were performed on top of the optimized B3LYP/def‐TZVP geometries. T_1_ diagnostics were run and match the observations of others,[^27^, ^28^, ^43^, ^44^] *i. e*., all species are borderline/show a slight multireference behavior. Increasing the basis set's size leads to more favorable T_1_ diagnostics.

In the evaluation of the several density functionals we performed single point energy calculations using the def2‐QZVPP basis set on top of geometries optimized using the same method but the def‐TZVP basis set.

For geometry optimizations we used energy convergence criteria of $10^{-7}$ E_h_ and gradient norms of at most $10^{-4}$ E_h_/a_0_. All reaction energies were calculated based on single‐molecule calculations. Vibrational and geometrical information originated always from the def‐TZVP calculations. The optimized structures were consistent with tabulated data.^[34]^

Besides requiring harmonic frequencies to estimate thermodynamic quantities, we used vibrational frequency analysis to confirm that the optimized structures corresponded to minima in the Potential Energy Surfaces.

All the harmonic frequencies used in this work are unscaled. We observed larger deviations between calculated and experimental frequencies for high‐frequency vibrational modes. These do not affect significantly the calculated thermodynamic properties at the temperatures of interest.

The statistical mechanical calculation of enthalpies, entropies and Gibbs energies was performed with the recently developed ULYSSES package.^[45]^ Thermodynamic quantities were evaluated using an interpolation of the free‐rotor with the harmonic oscillator, as originally defined by Grimme.^[46]^ A consistent partition function was furthermore used in the calculation of enthalpies. The interpolation is controlled by a single parameter, $\omega_{0}$ , which determines at which frequency the harmonic oscillator and the free‐rotor models mix. By default, we take $\omega_{0}$ =75 cm^−1^. Finally, all ideal gas properties are calculated using the standard temperature and pressure reference state.

Gibbs free energies of solvation in water were estimated using COSMOthermX19,^[47]^ program version 19.0.4 with the TZVPD‐FINE parametrization. For t‐N_2_O_4_, c‐N_2_O_4_ and ONONO there was previously no COSMO‐file available in the database. We ran the respective calculations using the B3LYP/def‐TZVP optimized geometries.

Plots were generated using python‘s matplotlib^[48]^ and the fits of thermodynamic functions were done using scipy‘s curve fit.^[49]^

## Conflict of interest

The authors declare no conflict of interest.

1

## Supporting information

As a service to our authors and readers, this journal provides supporting information supplied by the authors. Such materials are peer reviewed and may be re‐organized for online delivery, but are not copy‐edited or typeset. Technical support issues arising from supporting information (other than missing files) should be addressed to the authors.

Supporting Information

## Data Availability

The data that support the findings of this study are available from the corresponding authors upon reasonable request.
