# Supplementary material for: Acid Rain and Flue Gas: Quantum Chemical Hydrolysis of NO2
Source: Chemphyschem. 2022 Aug 16;23(21):e202200395. doi: 10.1002/cphc.202200395 (PMC9804303; doi:10.1002/cphc.202200395)
Supplement: Supplementary file 1 — Supporting Information [file CPHC-23-0-s001.pdf]

ChemPhysChem

Supporting Information

Acid Rain and Flue Gas: Quantum Chemical Hydrolysis of NO_2

Filipe Menezes* and Grzegorz Maria Popowicz*

Review Network Reactions involved in NO_2 Hydrolysis

The main reaction between NO_2 and H_2O is described by the reactions

$$\begin{aligned} 2NO_2(g) + H_2O(g) &\rightleftarrows HNO_3(g) + HNO_2(g) \\ 2NO_2(aq) + H_2O(l) &\rightleftarrows HNO_3(aq) + HNO_2(aq) \end{aligned} \tag{1}$$

where we considered that the reaction takes place either in bulk gas or in the bulk of an aqueous phase (meaning that NO_2 must first dissolve in water). Experimental evidence collected shows that reactions 1 only take place in aqueous solution, *i.e.* the gas phase reaction is non-spontaneous (*1*).

The two acids formed are particularly unstable species. For instance, nitrous acid may decompose to nitric acid and nitrogen oxide with additional release of water

$$\begin{aligned} 3HNO_2(g) &\rightleftarrows HNO_3(g) + 2NO(g) + H_2O(g) \\ 3HNO_2(aq) &\rightleftarrows HNO_3(aq) + 2NO(aq) + H_2O(l) \end{aligned} \tag{2}$$

and nitric acid decomposes to form nitrogen dioxide and oxygen (*2*)

$$\begin{aligned} 4HNO_3(g) &\rightleftarrows O_2(g) + 4NO_2(g) + 2H_2O(g) \\ 4HNO_3(aq) &\rightleftarrows O_2(aq) + 4NO_2(aq) + 2H_2O(l) \end{aligned} \tag{3}$$

The reaction is favoured at higher temperatures and it requires strongly acidic media.

In the presence of molecular oxygen, nitric oxide forms more nitrogen dioxide

$$NO(g) + \frac{1}{2}O_2(g) \rightleftarrows NO_2(g) \tag{4}$$

However, this latter reaction requires relatively high pressures and low temperatures to take place with considerable yield. For low- to intermediate pressures the oxidation of NO to NO_2 can be neglected from the whole network (*3, 4*). The reverse reaction is also possible (*5*), though only at extremely high temperatures.

One should furthermore consider the dimerization of nitrogen dioxide to form dinitrogen tetroxide (N_2O_4)

$$\begin{aligned} 2NO_2(g) &\rightleftarrows N_2O_4(g) \\ 2NO_2(aq) &\rightleftarrows N_2O_4(aq) \end{aligned} \tag{5}$$

which might take place in gas and aqueous phases (*6, 7*). Since the work of Abel *et al.* (*8*) it has been suggested the involvement of N_2O_4 in the hydrolysis of nitrogen dioxide. These

authors performed kinetic studies considering the reactions

$$\begin{aligned} N_2O_4(g) + H_2O(g) &\rightleftarrows HNO_2(g) + HNO_3(g) \\ N_2O_4(aq) + H_2O(aq) &\rightleftarrows HNO_2(aq) + HNO_3(aq) \end{aligned} \tag{6}$$

as alternatives to reaction 1.

The main argumentation for such suggestion was the molecularity of the reaction, but it was later also pointed out the expected higher physical solubility of N_2O_4 in water (in comparison to NO_2) (*1*). According to Henry constant compilation data (*9*), N_2O_4 is up to 100 times (physically) more soluble in water than is NO_2, though measurements related to the dimer are subject to the exact same uncertainties as the monomer. Nevertheless, data seems to favours the participation of dimers of NO_2 in the reaction for both gas and aqueous phases.

Additional pathways are opened by NO and NO_2, which may react to form dinitrogen trioxide and its isomer dioxodiazoxane

$$\begin{aligned} NO(g) + NO_2(g) &\rightleftarrows N_2O_3(g) \\ NO(aq) + NO_2(aq) &\rightleftarrows N_2O_3(aq) \end{aligned} \tag{7}$$

$$\begin{aligned} NO(g) + NO_2(g) &\rightleftarrows ONONO(g) \\ NO(aq) + NO_2(aq) &\rightleftarrows ONONO(aq) \end{aligned} \tag{8}$$

In the presence of water, the pair NO+NO_2, N_2O_3 or $ONONO$ may react to yield nitrous acid. The reaction of the former two to yield nitrous acid had already been suggest by Counce and Perona (*10*)

$$\begin{aligned} NO(g) + NO_2(g) + H_2O(g) &\rightleftarrows 2HNO_2(g) \\ NO(aq) + NO_2(aq) + H_2O(l) &\rightleftarrows 2HNO_2(aq) \end{aligned} \tag{9}$$

$$\begin{aligned} N_2O_3(g) + H_2O(g) &\rightleftarrows 2HNO_2(g) \\ N_2O_3(aq) + H_2O(l) &\rightleftarrows 2HNO_2(aq) \end{aligned} \tag{10}$$

$$\begin{aligned} ONONO(g) + H_2O(g) &\rightleftarrows 2HNO_2(g) \\ ONONO(aq) + H_2O(l) &\rightleftarrows 2HNO_2(aq) \end{aligned} \tag{11}$$

In order to account for the possible inhibition NO_2 at large partial pressures of NO, England and Corcoran (*11*) invoked the reactions

$$\begin{aligned} NO(g) + 2HNO_3(g) &\rightleftarrows 3NO_2(g) + H_2O(g) \\ NO(aq) + 2HNO_3(aq) &\rightleftarrows 3NO_2(aq) + H_2O(aq) \end{aligned} \tag{12}$$

Last but not least, the acid-base dissociation reactions for nitric and nitrous acids must also be taken in consideration.

$$HNO_3(aq) + H_2O(l) \rightleftarrows H_3O^+(aq) + NO_3^-(aq) \tag{13}$$

$$HNO_2(aq) + H_2O(l) \rightleftarrows H_3O^+(aq) + NO_2^-(aq) \tag{14}$$

Gibbs Free Energies for Reactions

Gibbs free energies for all reactions we studied are provided below. This concerns gas and aqueous phase data. Note that we also present the change in Gibbs free energy for the acid-base equilibria of nitric and nitrous acids. This data is not calculated, but instead collected from the literature. Reaction 3 (oxidation of NO by molecular oxygen) was not studied since we are interested in systems with low pressures. The equations we give are valid for the temperature range of 273.15 K and 373.15 K.

Table 1: Change in Gibbs free energies for the reactions of Figure 1 of the main text. Equations fitted for the temperature range 274.15-373.15 Kelvin. All fits with R^2 of at least 0.992, except for the case of reaction 8A', for which the linear fit leads to $R^2 = 0.695$.

	Reaction	$\Delta G(T)$
1G	$2NO_2(g) + H_2O(g) \rightleftarrows HNO_3(g) + HNO_2(g)$	$0.1451{\times}T - 41.513$
1A	$2NO_2(aq) + H_2O(l) \rightleftarrows HNO_3(aq) + HNO_2(aq)$	$0.1103{\times}T - 52.093$
2G	$3HNO_2(g) \rightleftarrows HNO_3(g) + 2NO(g) + H_2O(g)$	$-0.1250{\times}T + 37.815$
2A	$3HNO_2(aq) \rightleftarrows HNO_3(aq) + 2NO(aq) + H_2O(l)$	$-0.0938{\times}T + 37.809$
3	$NO(g) + \frac{1}{2}O_2(g) \rightleftarrows NO_2(g)$	not studied in this work.
4sG	$2NO_2(g) \rightleftarrows \text{s}{-}N_2O_4(g)$	$0.1683{\times}T - 55.810$
4sA	$2NO_2(aq) \rightleftarrows \text{s}{-}N_2O_4(aq)$	$0.1504{\times}T - 61.188$
4tG	$2NO_2(g) \rightleftarrows \text{t}{-}N_2O_4(g)$	$0.1256{\times}T - 28.327$
4tA	$2NO_2(aq) \rightleftarrows \text{t}{-}N_2O_4(aq)$	$0.0863{\times}T - 35.573$
4cG	$2NO_2(g) \rightleftarrows \text{c}{-}N_2O_4(g)$	$0.1260{\times}T - 17.660$
4cA	$2NO_2(aq) \rightleftarrows \text{c}{-}N_2O_4(aq)$	$0.0873{\times}T - 30.859$
5sG	$s\text{-}N_2O_4(g) + H_2O(g) \rightleftarrows HNO_2(g) + HNO_3(g)$	$-0.0232{\times}T + 14.297$
5sA	$s\text{-}N_2O_4(aq) + H_2O(aq) \rightleftarrows HNO_2(aq) + HNO_3(aq)$	$-0.0401{\times}T + 9.0952$
5tG	$t\text{-}N_2O_4(g) + H_2O(g) \rightleftarrows HNO_2(g) + HNO_3(g)$	$0.0196{\times}T - 13.185$
5tA	$t\text{-}N_2O_4(aq) + H_2O(aq) \rightleftarrows HNO_2(aq) + HNO_3(aq)$	$0.0241{\times}T - 16.520$
5cG	$c\text{-}N_2O_4(g) + H_2O(g) \rightleftarrows HNO_2(g) + HNO_3(g)$	$0.0191{\times}T - 23.853$
5cA	$c\text{-}N_2O_4(aq) + H_2O(aq) \rightleftarrows HNO_2(aq) + HNO_3(aq)$	$0.0230{\times}T - 21.233$
6G	$NO(g) + NO_2(g) \rightleftarrows N_2O_3(g)$	$0.1173{\times}T - 39.558$
6A	$NO(aq) + NO_2(aq) \rightleftarrows N_2O_3(aq)$	$0.0821{\times}T - 46.626$
6G'	$NO(g) + NO_2(g) \rightleftarrows ONONO(g)$	$0.1167{\times}T - 36.654$
6A'	$NO(aq) + NO_2(aq) \rightleftarrows ONONO(aq)$	$0.1002{\times}T - 36.664$
7G	$NO(g) + NO_2(g) + H_2O(g) \rightleftarrows 2HNO_2(g)$	$0.1351{\times}T - 39.664$
7A	$NO(aq) + NO_2(aq) + H_2O(l) \rightleftarrows 2HNO_2(aq)$	$0.1021{\times}T - 44.951$
8G	$N_2O_3(g) + H_2O(g) \rightleftarrows 2HNO_2(g)$	$0.0178{\times}T - 0.1062$
8A	$N_2O_3(aq) + H_2O(l) \rightleftarrows 2HNO_2(aq)$	$0.0199{\times}T + 1.6751$
8G'	$ONONO(g) + H_2O(g) \rightleftarrows 2HNO_2(g)$	$0.0183{\times}T - 3.0102$
8A'	$ONONO(aq) + H_2O(l) \rightleftarrows 2HNO_2(aq)$	$0.0019{\times}T - 8.2867$
9G	$NO(g) + 2HNO_3(g) \rightleftarrows 3NO_2(g) + H_2O(g)$	$-0.1552{\times}T + 43.3613$
9A	$NO(aq) + 2HNO_3(aq) \rightleftarrows 3NO_2(aq) + H_2O(aq)$	$-0.1186{\times}T + 59.2344$
10	$HNO_3(aq) + H_2O(l) \rightleftarrows H_3O^+(aq) + NO_3^-(aq)$	$-0.003178{\times}RT$ (*12*)
11	$HNO_2(aq) + H_2O(l) \rightleftarrows H_3O^+(aq) + NO_2^-(aq)$	$0.007714{\times}RT$ (*13*)

Formation and Hydrolysis of N_2O_4 conformers

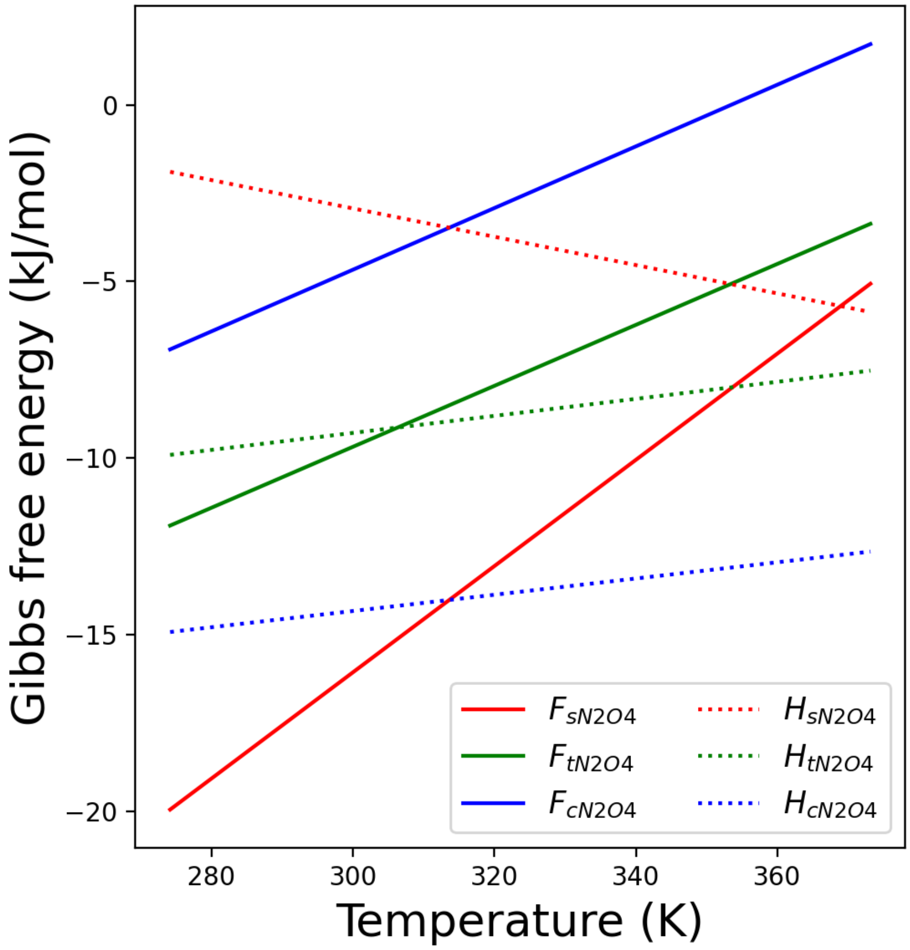

Figure 1: Comparison of Gibbs free energies for the formation and hydrolysis of several conformers of dinitrogen tetroxide in water.

Approximations in Setting Up Thermodynamic System

Because we are interested in the thermodynamic equilibrium, all transient species are neglected. This includes, *e.g.*, dimers of NO_2, N_2O_3 and $ONONO$.

As is evidenced by experimental data, NO_2 does not react in the gas phase. There are however some gas phase reactions that might be of relevance in certain extreme conditions, like low pH. We considered systems which are reasonably dilute in water, and in our conditions the lowest pH attainable is around 5. At such large pH value, the concentration

of undissociated nitrous acid is very low, which severely hinders the eventual gas phase processes. Under such conditions, their contribution to the equilibrium is minor and we neglected it altogether. We note that including such gas phase processes would require additional Henry constants for other species. This poses an even larger burden in solving the equilibrium, as more processes hinder convergence (from the mathematical point of view) or, according to actual experimental observations, the establishment of equilibrium may itself be inhibited (*11*).

Acids were not considered in the gas phase. This is partially related to what was exposed in the previous paragraph, but also related to the quite favourable Gibbs free energies of solvation for those species. One is then to expect a preponderance of those species in the aqueous solution. According to our COSMOtherm calculations, the Gibbs free energy of solvation for nitric acid is limited to the interval -5.05 and -6.16 $kcal/mol$ for temperatures between 274.15 - 373.15 K. For nitrous acid the respective solvation data lies between -3.2 and -4.3 $kcal/mol$ for the same temperature range.

We assume that the gas phase is constantly saturated with water and there will always be enough water to form the liquid phase. This has mainly two implications. On one hand, the consumption/solvation of gases in liquid water will not create a "vacuum" in the gas phase (stability). Because there is an excess of water, the hydrolysis reaction will barely affect the total concentration of liquid water in the system. This we did not neglect in our actual calculations to verify the validity of the approximation (another section of Supporting Information). Consequently, the number of moles of water in each phase will only be a function of temperature and we can neglect the vapour-liquid equilibrium of water.

Though in real systems one cannot exclude the presence of oxygen, we completely neglect this species and its effect in oxidizing nitric oxide to nitrogen dioxide. This is because we focus on low-pressure conditions, for which the oxidation reaction has negligible yields (*10*), and because oxygen does not affect the hydrolysis of nitrogen dioxide in any other way (*11*). Last but not least, we assume that the nitrite formed is stable and will not decompose at any conditions. Though that is realistic for the pH values of interest for us (*14*), it may no longer be the case at higher temperatures (*15*).

Because we start our calculations assuming nitrogen oxides in a gas phase (which are in a first instance dissolved in water), Henry constants or Gibbs free energies for the physical solvation of the nitrogen oxides in water are required. Though that information is readily available for NO at some temperatures, the same does not apply for nitrogen dioxide. To model the dissolution of gases in water we used the temperature dependent

COSMOtherm results. The solvation of NO in water is described by (units of kJ/mol)

$$\Delta_{solv}G^{NO} = -0.0001889T^2 + 0.15769T - 24.099 \quad (15)$$

whereas for NO_2 we fitted

$$\Delta_{solv}G^{NO2} = -0.0001816T^2 + 0.15491T - 25.902 \quad (16)$$

Technically speaking we could have treated NO's solubility in water using fits to experimental data. We wanted however to use consistent data sources for all similar processes we simulated. This has furthermore the advantage that the calculated $\Delta_{solv}G$ for NO can be somehow extrapolated for temperatures other than 273.15-373.15 K, to include, *e.g.* pressure effects [1].

Acid-base constants for nitric and nitrous acids were however taken from experimental data. This is because quantum chemical and solvation methods are still too imprecise to accurately pK_a data without recurrence to additional empirical correlations.

Algorithm Details

Using the above assumptions and calculated thermodynamic data, we built a python script that calculates equilibrium concentrations of nitrogen oxides, acids and their conjugate bases. This script takes as input the initial mole numbers of NO_x in the gas phase and the total number of moles of liquid water. Note that we assume as starting conditions that the water phase is pure and that dissolved gases come as equilibrium is established. We take a target pH value as an additional input variable. Though it might be seen as an additional restriction to aid convergence, physical meaning may be attributed to it though. This can be interpreted as a pH control in industrial setups or, in atmospheric chemistry, as the presence of a pH buffer like carbon dioxide. Other physical variables are temperature, volume and pressure.

Despite the freedom in setting up a target pH value as argument, the limits for pH are defined by the system itself. This is because i) for each amount of nitrogen dioxide there is a maximum acidity achievable (no acid is added) and attempts to lower excessively the pH result in mathematical domain error; ii) there are convergence issues at too large values of pH due to the (excessively) low concentration of nitric acid, which also hinders convergence. If we take for instance a test-case with initially $5{\times}10^{-5}$ mol of $NO_2(g)$,

[1] Though unreasonable for experimental data, modelled such as what we provided is well behaved.

$5{\times}10^{-4}$ *mol* of $NO(g)$, 0.5 *mol* of $H_2O(l)$ at 50 *Celsius* and $pH = 9$, the system of equations does not converge and the amount of undissociated nitric acid is below $1{\times}10^{-16}$ *mol*.

Upon setting initial conditions, the script calculates the equilibrium constants for each reaction and for every iteration each equilibrium is solved until residuals are below a certain threshold in two consecutive iterations. This had to be done to avoid premature exit of the iterative cycle due to very low unconverged concentrations. For convenience we decided to define the residuals as the square of the difference between equilibrium constant and reaction quotient. For instance, the contribution of reaction 1A to the total residuals is

$$\left(K(T) - \frac{a[HNO_2(aq)]a[HNO_3(aq)]}{a[H_2O(l)]a[NO_2(aq)]^2}\right)^2 \tag{17}$$

where $a[X]$ is the activity of species X. To keep the model simple we assumed that all activity coefficients are close to unit. This approximation is particularly poor for the anions, when at higher concentrations. To estimate the maximum error we incur by such an approximation, we double checked the Debye-Hückel activity coefficients for the anions (*16*). In the worst case scenario where the total number of moles of both ions is 0.001 *mol* activity coefficients are 0.8. For temperature and pH studies where we have ten times less ions, the activity coefficients are larger than 0.9, meaning that deviations are more amenable. COSMOtherm calculations we did on water showed that the approximation should be significantly better: the largest deviation expected in activity coefficients is by 0.07. In productive calculations we would strongly recommend not neglecting activity coefficients.

In all reactions equilibrated, there was a need to solve up to quartic polynomials. In doing so we took advantage of numpy's root finder (*17*), which greatly simplified the task.

Verification of Approximations

Though absent in any of the plots, we verified that the mole fraction of water is for numerically constant. The largest change in the concentration of water between initial conditions and equilibrium is 0.5 %. The concentration of actual nitric acid in water (and not nitrate) at equilibrium is always below the threshold value of $2{\times}10^{-11}$ *mol*, irrespective of the initial conditions. Typically the concentration of the acid is lower. For nitrous acid we verify a maximum of 10^{-6} *mol* for the calculations at lowest pH. Even if we were to consider only the worst case scenario for nitrous acid and use the solvation

energy at 100 Celsius at the largest temperature herein studied (80 Celsius), the amount of the species in the gas phase would be at least 100 times less than it is in water. We conclude that our calculations on the equilibrated systems are in agreement with the underlying assumptions.

Geometries and CCSD(T)/CBS energies

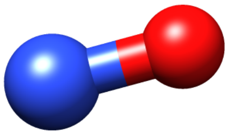

Figure 2: Molecule NO.

```
2
E(CCSD(T)/CBS) = -129.78778868843526
N    -5.7007     2.3426     -0.2157
O    -4.5963     2.6403     -0.1246
```

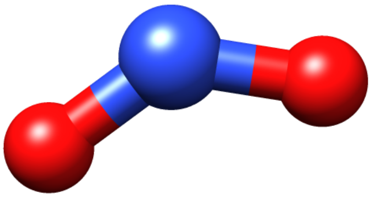

Figure 3: Molecule NO_2.

```
3
E(CCSD(T)/CBS) = -204.91884705420986
N     -5.7025     2.2976     -0.1094
O     -4.5708     2.6705     -0.1783
O     -6.3492     1.7494      0.7307
```

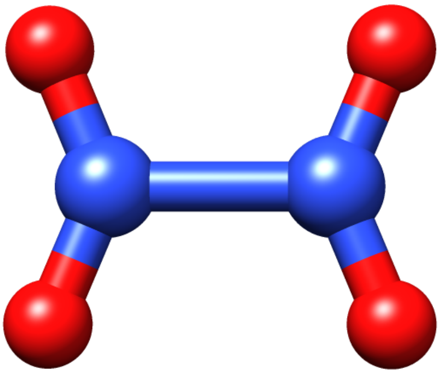

Figure 4: Molecule s-N_2O_4.

6			
E(CCSD(T)/CBS) = -409.8640984445898			
N	-0.8517	1.4176	0.1539
N	0.8977	1.0620	-0.1539
O	-1.3475	2.0623	-0.7116
O	-1.2409	0.9529	1.1751
O	1.2869	1.5267	-1.1751
O	1.3935	0.4173	0.7116

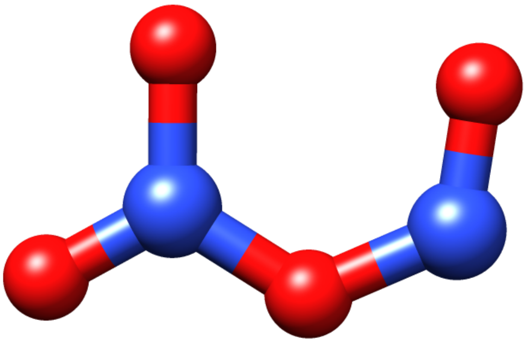

Figure 5: Molecule c-N_2O_4.

6			
E(CCSD(T)/CBS) = -409.847889381902			
N	-2.86970	2.93628	0.00000
O	-3.90852	3.75972	0.00000
O	-1.74245	3.33041	0.00000
N	-3.81709	5.08220	0.00000

O	-4.81616	5.73355	0.00000
O	-2.65188	5.69537	0.00000

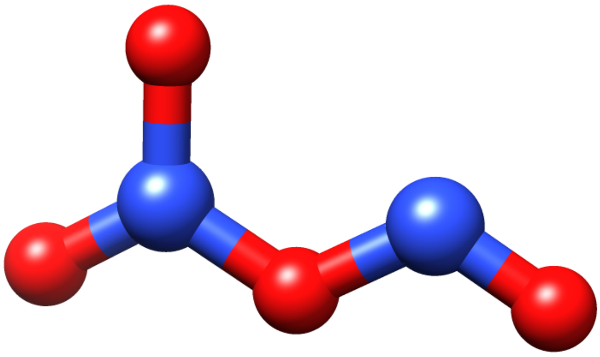

Figure 6: Molecule t-N_2O_4.

6

E(CCSD(T)/CBS) = -409.851896550638

N	-3.08578	2.79286	0.00000
O	-4.05001	3.68976	0.00000
O	-3.35252	1.63043	0.00000
N	-3.78117	4.98118	0.00000
O	-4.67587	5.76982	0.00000
O	-2.53596	5.40929	0.00000

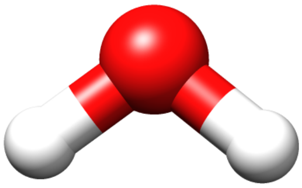

Figure 7: Molecule H_2O.

3

E(CCSD(T)/CBS) = -76.38327108185337

O	-4.9830	-0.6460	-1.8093
H	-4.0206	-0.6114	-1.8239
H	-5.2684	0.2015	-2.1672

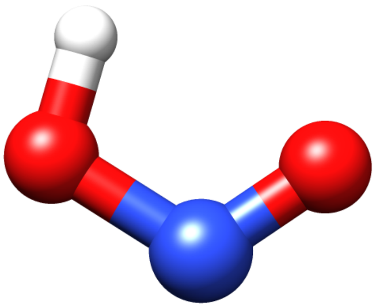

Figure 8: Molecule c-HNO_2.

```
4
E(CCSD(T)/CBS) = -205.55463120729823
N      -5.8678      2.2997      -0.2343
O      -4.7484      2.5660      -0.0578
O      -6.4338      1.6455       0.9172
H      -7.3496      1.4835       0.6394
```

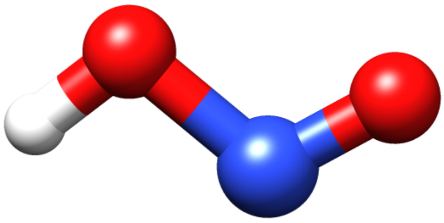

Figure 9: Molecule t-HNO_2.

```
4
E(CCSD(T)/CBS) = -205.554286549141
N      -5.7323       2.3477      -0.2506
O      -4.6044       2.5901      -0.0106
O      -6.4021       1.7067       0.7941
H      -5.7483       1.6022       1.5185
```

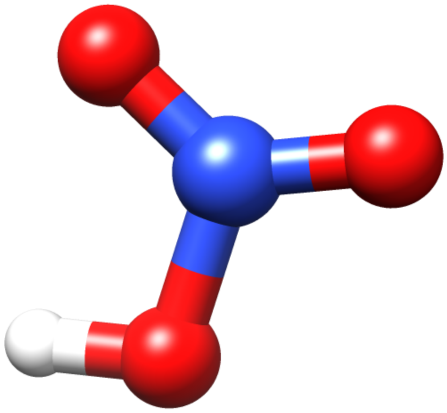

Figure 10: Molecule HNO_3.

```
5
E(CCSD(T)/CBS) = -280.68797380061216
N      -5.9532      2.3271      -0.3361
O      -4.8084      2.5620      -0.0943
O      -6.3768      2.7807      -1.6172
H      -7.3122      2.5132      -1.6482
O      -6.7981      1.7716       0.3278
```

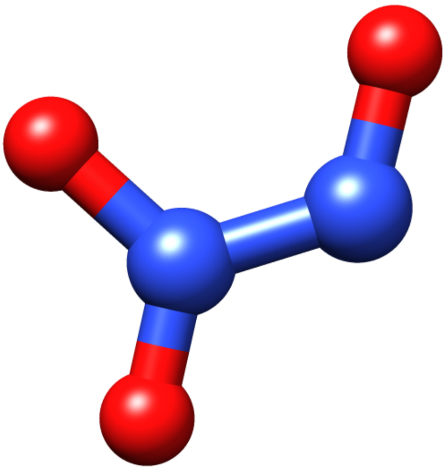

Figure 11: Molecule N_2O_3.

```
5
E(CCSD(T)/CBS) = -334.7252940651495
N          -3.08706          2.77640          0.00000
O          -4.06186          3.66098         -0.00000
```

O	-3.34516	1.61199	0.00000
N	-1.77510	3.18357	0.00000
O	-1.49709	4.34468	-0.00000

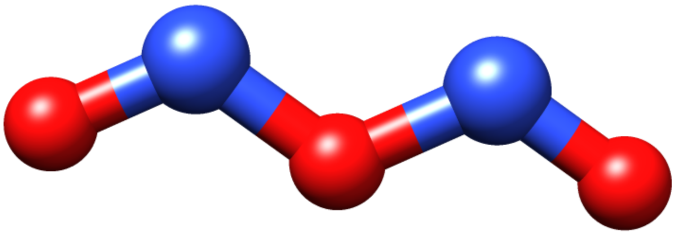

Figure 12: Molecule $ONONO$.

5

E(CCSD(T)/CBS) = -334.72268341184696

N	-3.09505	2.80760	0.00000
O	-4.07523	3.68352	-0.00000
O	-3.33854	1.64005	0.00000
N	-3.80722	4.97044	0.00000
O	-4.69676	5.76490	0.00000

Bibliography

1. M. T. C. Martins-Costa, J. M. Anglada, J. S. Francisco, and M. F. Ruiz-López, "The aqueous surface as an efficient transient stop for the reactivity of gaseous NO_2 in liquid water," *Journal of the American Chemical Society*, vol. 142, no. 50, pp. 20937–20941, 2020.

2. G. D. Robertson, D. M. Mason, and W. H. Corcoran, "The kinetics of the thermal decomposition of nitric acid in the liquid phase," *The Journal of Physical Chemistry*, vol. 59, no. 8, pp. 683–690, 1955.

3. T. Ting, R. Stanger, and T. Wall, "Laboratory investigation of high pressure NO oxidation to NO_2 and capture with liquid and gaseous water under oxy-fuel CO_2 compression conditions," *International Journal of Greenhouse Gas Control*, vol. 18, p. 15, 2013.

4. V. White, L. Torrente-Murciano, D. Sturgeon, and D. Chadwick, "Purification of oxyfuel-derived CO_2," *Energy Procedia*, vol. 1, no. 1, pp. 399–406, 2009. Greenhouse Gas Control Technologies 9.

5. D. M. Yost and H. Russell, *Systematic Inorganic Chemistry.* Prentice-Hall, 1944.

6. M. Grätzel, A. Henglein, J. Lilie, and G. Beck, "Pulsradiolytische untersuchung einiger elementarprozesse der oxydation und reduktion des nitritions," *Berichte der Bunsengesellschaft für physikalische Chemie*, vol. 73, no. 7, pp. 646–653, 1969.

7. A. Treinin and E. Hayon, "Absorption spectra and reaction kinetics of NO_2, N_2O_3, and N_2O_4 in aqueous solution," *Journal of the American Chemical Society*, vol. 92, no. 20, pp. 5821–5828, 1970.

8. E. Abel, H. Schmid, and E. Roemer, "Kinetik der salpetrigen saeure VII. geschwindigkeit und temperatur," *Zeitschrift fuer physikalische Chemie A*, vol. 148, no. 5, p. 337, 1930.

9. R. Sander, "Compilation of henry's law constants (version 4.0) for water as solvent," *Atmospheric Chemistry and Physics*, vol. 15, no. 8, pp. 4399–4981, 2015.

10. R. M. Counce and J. J. Perona, "Scrubbing of gaseous nitrogen oxides in packed towers," *AIChE Journal*, vol. 29, no. 1, pp. 26–32, 1983.

11. C. England and W. H. Corcoran, “Kinetics and mechanisms of the gas-phase reaction of water vapor and nitrogen dioxide,” *Industrial & Engineering Chemistry Fundamentals*, vol. 13, no. 4, pp. 373–384, 1974.

12. J. A. Dean, *Lange's Handbook of Chemistry.* McGraw-Hill, 13 ed., 1985.

13. M. J. O'Neil, *The Merck index: An encyclopedia of chemicals, drugs, and biologicals.* Merck and Co., Inc., 13 ed., 2006.

14. W. Braida and S. K. Ong, “Decomposition of nitrite under various ph and aeration conditions,” *Water, Air, and Soil Pollution*, vol. 118, no. 1, p. 13, 2000.

15. F. Winkler, N. Schoedel, H.-J. Zander, and R. Ritter, “Cold denox development for oxyfuel power plants,” *International Journal of Greenhouse Gas Control*, vol. 5, p. s231, 2011.

16. J. Kielland, “Individual activity coefficients of ions in aqueous solutions,” *Journal of the American Chemical Society*, vol. 59, no. 9, pp. 1675–1678, 1937.

17. C. R. Harris, K. J. Millman, S. J. van der Walt, R. Gommers, P. Virtanen, D. Cournapeau, E. Wieser, J. Taylor, S. Berg, N. J. Smith, R. Kern, M. Picus, S. Hoyer, M. H. van Kerkwijk, M. Brett, A. Haldane, J. F. del Río, M. Wiebe, P. Peterson, P. Gérard-Marchant, K. Sheppard, T. Reddy, W. Weckesser, H. Abbasi, C. Gohlke, and T. E. Oliphant, “Array programming with NumPy,” *Nature*, vol. 585, no. 7825, pp. 357–362, 2020.
